# Foreign Body Reaction Following Achilles Tendon Reconstruction With the Ligament Advanced Reconstructive System: Patient Outcomes and Clinical Course

**DOI:** 10.7759/cureus.48686

**Published:** 2023-11-12

**Authors:** Niall Fitzpatrick, Theenesh Balakrishnan, Anand Pillai

**Affiliations:** 1 Trauma and Orthopaedics, Wythenshawe Hospital, Manchester University NHS Foundation Trust, Manchester, GBR

**Keywords:** lars ligament, lars, achilles tendon injury, foreign body reaction, granuloma, ligament reconstruction, ligament advanced reconstructive system

## Abstract

The Ligament Advanced Reinforcement System (LARS) is a common choice for ligament reconstruction in the lower limb due to its good functional and quality of life (QoL)-related outcomes. It is commonly used for Achilles tendon repair following a rupture. While it facilitates tissue ingrowth and boasts good biocompatibility, we report on multiple cases whereby foreign body reactions have led to the growth of granulomas requiring surgical excision and Flexor Hallucis Longus (FHL) transfer. Following these cases, patients have been shown to have excellent functional and QoL-related outcomes using the Manchester Oxford Foot Questionnaire (MOX-FQ) and Foot and Ankle Ability Measure (FAAM). Surgeons should consider FHL transfer as an alternative in patients undergoing Achilles tendon repair and be aware of the risk of foreign body reactions and the impact on ankle function and QoL post-operatively.

## Introduction

The Achilles tendon is the largest tendon in the human body, with the highest strength. It is also, however, the most commonly ruptured tendon in the lower extremity, commonly occurring in the young to middle-aged population. Most frequently, this is due to sporting injuries caused by excessive loading [1]. Achilles tendon rupture has an annual incidence of approximately 18 per 100’000 people, with around 4’500 patients seeking medical help for rupture annually in the United Kingdom [2-3]. The optimal management of Achilles tendon rupture is debated, with surgical repair and conservative treatment conferring a different range of benefits and complications. Of note, surgical repair is associated with a reduced risk of re-rupture. However, its complication profile includes nerve injury, adhesion, infection, and foreign body reactions, depending on the repair method [4].

The Ligament Advanced Reinforcement System (LARS) is a synthetic, non-absorbable ligament comprising polyethylene terephthalate fibres (PET) composed of two parts: an intraosseous segment composed of longitudinal fibres bound together by a transverse knitted structure and an intra-articular segment composed of parallel longitudinal free fibres twisted at 90° [5]. LARS has been shown to provide strength and stability in ligament repair and reconstruction and was promoted as an alternative to autologous grafts and the resultant donor site morbidity, alongside lower rates of failure and synovitis compared to alternative ligament repair systems such as Dacron, Gore-Tex, and Trevira [6]. In-vitro research has demonstrated the facilitation of the in-growth of fibroblasts and tenocytes, improving biocompatibility in the augmentation or repair of native tissue [7-8]. Traditionally, LARS has been popular, with low complication rates, ease of commercial availability, and good functional and quality-of-life-related results [9]. There are limited studies describing granulomatous inflammation in response to tendon reconstruction using LARS; however, this paper focuses on three cases where excision and in-situ reconstruction using the Flexor Hallucis Longus (FHL) tendon were required.

## Case presentation

Case 1

**Figure 1:**
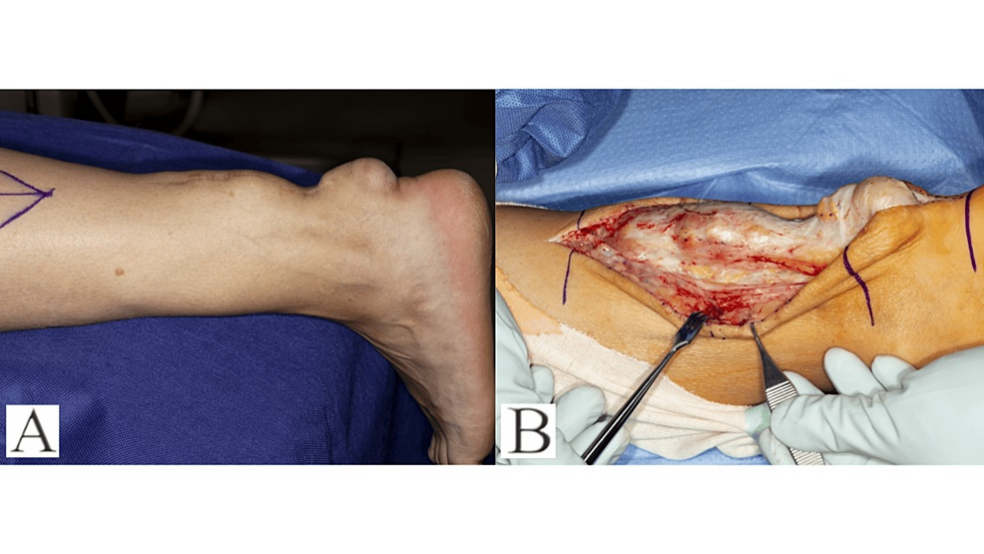
(A-B) Pictures representing lobular mass overlying the heel pre-operatively and intra-operatively prior to excision

**Figure 2:**
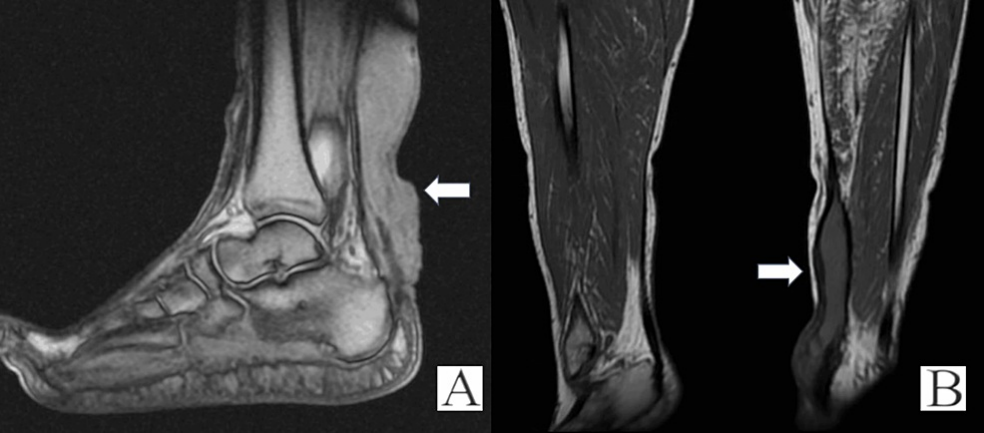
(A-B) T2 weighted sagittal MRI showing distinct masses in Achilles tendon

A 59-year-old male sustained a traumatic rupture of the Achilles tendon while playing soccer. Initially, they were treated in an Equinus cast for a period of six weeks. Following the removal of the cast and his return to work, he sustained a further injury while walking on uneven ground two weeks later. They underwent surgical repair using the LARS system. Following a period of physiotherapy, the patient reported no functional limitations relating to his foot or ankle and returned to work. Thirteen years post-operatively, they report that swelling began to occur at the surgical site, which continued to grow over a period of seven years (Figure 1A-1B). There were no issues with either deep or superficial infections. This patient sought an orthopaedic opinion as a result of difficulty wearing conventional footwear. MRI revealed a 15 cm × 3 cm × 3 cm lobulated mass (Figure 2A-2B).

Case 2

This 68-year-old male sustained a forced ankle dorsiflexion injury while diving into a swimming pool. The diagnosis of Achilles tendon rupture was delayed, and at the time of diagnosis, there was significant tendon retraction. They were initially treated surgically using the LARS system. Post-operatively, there were no functional limitations, and the patient returned to their functional baseline, recommending an active lifestyle. Two years post-operatively, they reported swelling at the surgical site without any functional limitations. This swelling expanded over a period of 18 months prior to the orthopaedic referral. The large cystic swelling did not demonstrate evidence of infection. MRI demonstrated a 10 cm × 3.3 cm × 3cm collection within the Achilles tendon, reported to demonstrate an evolving haematoma.

Case 3

A 69-year-old male who sustained a traumatic rupture of the Achilles tendon during a paraglider landing was presented. This traumatic Achilles tendon rupture was treated in an Equinus cast for six weeks, followed by full mobilisation. He sustained a re-rupture one year later while walking and underwent repair using the LARS system. This patient reported a full return to functionality, including his ability to run long distances. Again, there was a delay in the swelling. This was first noticed ten years after Achilles tendon repair. Over the course of five years, this increased in size while not impacting the function of his foot or ankle. He sought an orthopaedic opinion again as a result of difficulties with footwear, in which he described having to remove the heel counter from shoes in order to fit his foot inside. Again, there were no issues relating to either a deep or superficial infection. An MRI scan revealed a 16 cm × 6 cm × 3 cm heterogenous collection within the Achilles tendon.

Differential diagnoses in cases of such a swelling overlying Achilles tendon include chronic tendon ruptures, mid-substance tendinopathy, and benign lesions, including lipoma or ganglion cysts. In the case of those having undergone previous Achilles tendon repair using the LARS system, foreign body granuloma, abscess, and infection were considered to be more likely. In each case, surgical excision and analysis of excised material were required for histological diagnosis.

Surgical technique

All three patients underwent excision of the Achilles tendon pseudotumour and FHL transfer. All were approached via a posterior midline incision using the site of the previous scar. Skin flaps were raised, allowing visualisation of the pseudotumour, alongside visualisation and protection of the short saphenous vein and sural nerve. Tendoachilles/pseudotumour was excised proximally at the transverse intermuscular septum and distally at the calcaneum. The excised tendons were sent for histological analysis. FHL tendon harvest was performed in each case, with attachment to the calcaneum via the G-Force tenodesis system and JuggerKnot all-suture anchors. An Equinus cast and PICO dressing were applied in each case, with instructions for a period of non-weight bearing post-operatively.

Results

**Figure 3:**
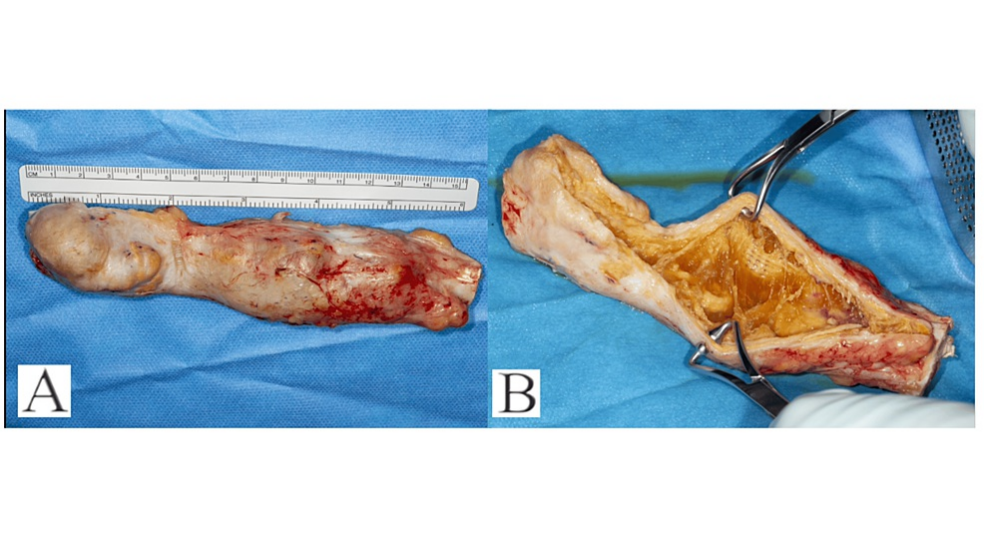
(A-B) Intra-operative imaging demonstrating excised tendon (A) and synthetic fibrous strands internally

**Figure 4:**
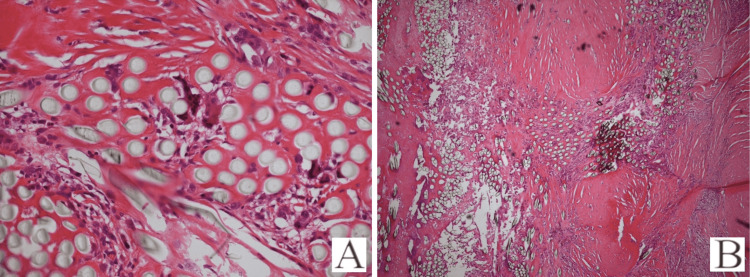
(A-B) Histological slides from excised tissue (A) High magnification slide demonstrating amorphous, eosinophilic, acellular LARS ligament (white bundles), with foreign body giant cells and foamy histiocytes; surrounding hypocellular fibrous tissue; (B) low magnification slide demonstrating foreign body giant cell response to suture material with fibrous tissue ingrowth. Images used with permission of Henry et al., the case report written by whom represents one of the patients in this series [10].

Initial assessment of excised material demonstrated clearly visible synthetic fibrous strands with surrounding granulomatous tissue, consistent with foreign body granuloma (Figure 3A-3B). Macroscopically, all three samples demonstrated fibrous ingrowth. Histological analysis of excised tendons demonstrated a foreign body response in each case, with the presence of multinucleated histiocytes (Giant cells) associated with inflammation (Figure 4A-4B).

Outcomes

Patient-reported outcomes were measured via the Foot and Ankle Ability Measure (FAAM) Activities of Daily Living (ADL) subscale and the Manchester Oxford Foot Questionnaire (MOX-FQ), both independently validated scoring systems for health-related quality of life (QoL) and ankle functionality. MOF-FQ has been validated for the impact of intervention following operative intervention [11]. FAAM provides a validated measure of physical function for individuals with a range of musculoskeletal disorders, including those of the foot and ankle [12]. Both patient-reported outcome measures (PROMS) questionnaires are scored using a five-point Likert scale. Two patients were contacted for questionnaire completion, with a mean time from surgery of 63.5 months (range 21-55).

A higher score in MOX-FQ correlates to a worse function of the joint (scored out of 64 points), while a higher score in FAAM (scored out of 84) reflects a higher level of physical function. The average score in the study cohort was 75.5 (range 69-82) in the FAAM questionnaire, with an average score of five (range 2-10) in MOX-FQ scoring with evidence of strong surgical outcomes across the pain, walking/standing, and social interaction domains. Overall, these indicate excellent physical function post-operatively. One patient reported no limitation in the pain and social interaction subscales on the MOX-FQ PROMS questionnaire, while their only consideration was being conscious of causing discomfort when walking on rough surfaces. Another patient described occasional mild pain in his ankle in the evenings. Neither had any functional limitations following their surgery.

Analysis of the PROMS scoring of the FAAM Activities of Daily Living subscale demonstrated excellent outcomes following pseudotumour excision and FHL transfer, with one patient reporting no limitation of difficulties, including heavy work; the other patient scored 63/84 points, with his limitations described as an inability to stand on his toes and squat following the FHL transfer replacement of the Achilles tendon following pseudotumour excision. Other limitations included ‘slight’ limitations to heavy work such as climbing or pushing/pulling items and ‘slight’ difficulty traversing stairs. On further discussion, these caused no functional limitations to his day-to-day living. At the time of writing, the third patient included in the series was still in a protective cast, so the above scales were not applicable.

## Discussion

The surgical approach to the repair of ruptured tendons provides a reconstructive conundrum. Autografts have limitations, for example, the variation of properties and limited dimensions of the donor tendon, alongside the initial loss of strength during the necrotic stage [13]. Synthetic ligaments provided an alternative, providing immediate tensile strength in repair without the need for donor harvesting. Artificial substitutes were popularised for ACL repair in the 1970s and 1980s; however, these were associated with high rates of mechanical breakdown, synovitis, and ultimately a high failure rate [14]. Laboratory analysis has demonstrated the role of wear microparticles in leading to a local inflammatory response. These microparticles have been shown to result in synovitis and have an impact on cartilage degeneration [15]. In vivo analysis has also demonstrated poor cytocompatibility, leading to loosening, mechanical failure, and synovitis of the knee joint as a result of wear particles [16]. Advancements in the technology associated with manufacturing led to ligament repair systems such as LARS and Ortho-tape, both consisting of woven PET fibres, designed to mimic the internal architecture of native ligaments. While these are non-absorbable, they permit donor ingrowth of fibroblasts and tenocytes, aiming to improve biological compatibility [8].

There are limited reports of the development of complications relating to foreign body reactions with the LARS system for Achilles tendon repair. Studies of ligament reconstruction using LARS have primarily focused on ACL repair. A recent prospective study from Smolle et al. into long-term clinical and QoL-related outcomes in LARS treatment of ACL tears showed good functional and QoL-related results but a high complication rate (66%, n=27). These included graft failure in 24% (n=10) of patients and reactive synovitis in 20% (n=8) [9].

There are limited resources detailing the outcomes of Achilles tendon repair using LARS, with two case reports of foreign body reactions leading to a granuloma that required resection [10,17]. This case series demonstrates that resection of the previously-repaired Achilles tendon with reconstruction via Flexor Hallucis Longus transfer is highly efficacious, with patients demonstrating a full recovery to their mobility baseline and functionality, with a return of functional mobility noted following the period of post-operative immobilisation. This case series provides reason to be cautious in the use of synthetic ligaments over the use of autografts in chronic Achilles tendon rupture, with FHL transfer demonstrating high efficacy in this small study cohort. In cases where surgical reconstruction is clinically indicated, repair using FHL transfer is an appropriate alternative to the use of a synthetic LARS ligament repair system. Patient-reported outcomes demonstrate a full return to function in patients who underwent excision of pseudotumour and FHL transfer as a result of foreign body reaction, with this small cohort not demonstrating any major complications or limitations of their ADLs post-operatively.

## Conclusions

While the synthetic option is easily accessible, research shows that it lacks real biocompatibility; hence, FHL transfer should be taken into consideration over the use of LARS ligament in patients deemed suitable for surgical intervention in the repair of severe Achilles tendon rupture. No high-quality prospective trials evaluating outcomes or complication rates after Achilles tendon replacement utilizing the LARS ligament repair technique were found in the literature review. Foreign body reactions must be taken into consideration in cases where oedema surrounds the site of previous ligament reconstruction utilizing the LARS technique, with FHL transfer being a suitable option for reconstruction.

## References

[1] Mattila VM, Huttunen TT, Haapasalo H, Sillanpää P, Malmivaara A, Pihlajamäki H (2015). Declining incidence of surgery for Achilles tendon rupture follows publication of major RCTs: evidence-influenced change evident using the Finnish registry study. Br J Sports Med.

[2] Uquillas CA, Guss MS, Ryan DJ, Jazrawi LM, Strauss EJ (2015). Everything Achilles: knowledge update and current concepts in management: AAOS exhibit selection. J Bone Joint Surg Am.

[3] Boyd RP, Dimock R, Solan MC, Porter E (2015). Achilles tendon rupture: how to avoid missing the diagnosis. Br J Gen Pract.

[4] She G, Teng Q, Li J, Zheng X, Chen L, Hou H (2021). Comparing surgical and conservative treatment on Achilles tendon rupture: a comprehensive meta-analysis of RCTs. Front Surg.

[5] Parchi PD, Ciapini G, Paglialunga C (2018). Anterior cruciate ligament reconstruction with Lars artificial ligament-clinical results after a long-term follow-up. Joints.

[6] Batty LM, Norsworthy CJ, Lash NJ, Wasiak J, Richmond AK, Feller JA (2015). Synthetic devices for reconstructive surgery of the cruciate ligaments: a systematic review. Arthroscopy.

[7] (2023). LARS™. https://www.coringroup.com/healthcare-professionals/solutions/lars/.

[8] Trieb K, Blahovec H, Brand G, Sabeti M, Dominkus M, Kotz R (2004). In vivo and in vitro cellular ingrowth into a new generation of artificial ligaments. Eur Surg Res.

[9] Smolle MA, Fischerauer SF, Zötsch S (2022). Long-term outcomes of surgery using the Ligament Advanced Reinforcement System as treatment for anterior cruciate ligament tears. Bone Joint J.

[10] Henry J, Konarski AJ, Joseph L, Pillai A (2018). Foreign body reaction with granuloma following Achilles tendon reconstruction with the LARS ligament. J Surg Case Rep.

[11] Morley D, Jenkinson C, Doll H, Lavis G, Sharp R, Cooke P, Dawson J (2013). The Manchester-Oxford Foot Questionnaire (MOXFQ): development and validation of a summary index score. Bone Joint Res.

[12] Martin RL, Irrgang JJ, Burdett RG, Conti SF, Van Swearingen JM (2005). Evidence of validity for the Foot and Ankle Ability Measure (FAAM). Foot Ankle Int.

[13] Wang XM, Ji G, Wang XM, Kang HJ, Wang F (2018). Biological and biomechanical evaluation of autologous tendon combined with ligament Advanced Reinforcement system artificial ligament in a rabbit model of anterior cruciate ligament reconstruction. Orthop Surg.

[14] Savarese A, Lunghi E, Budassi P (1993). Remarks of the complications following ACL reconstruction using synthetic ligaments. Ital J Orthop Traum.

[15] Olson EJ, Kang JD, Fu FH, Georgescu HI, Mason GC, Evans CH (1988). The biochemical and histological effects of artificial ligament wear particles: in vitro and in vivo studies. Am J Sports Med.

[16] Mao Z, Fan B, Wang X (2021). A systematic review of tissue engineering scaffold in tendon bone healing in vivo. Front Bioeng Biotechnol.

[17] Niazi N, Aljawadi A, Khan M (2022). Foreign body reaction with granuloma 10 years after Achilles tendon reconstruction with the Lars ligament. Int J Regen Med.

